# Prediction of lymphovascular invasion of gastric cancer based on contrast-enhanced computed tomography radiomics

**DOI:** 10.3389/fonc.2024.1389278

**Published:** 2024-09-05

**Authors:** Si-Yu Zhen, Yong Wei, Ran Song, Xiao-Huan Liu, Pei-Ru Li, Xiang-Yan Kong, Han-Yu Wei, Wen-Hua Fan, Chang-Hua Liang

**Affiliations:** ^1^ Department of Radiology, Xinxiang Medical University First Affiliated Hospital, Xinxiang, China; ^2^ Henan Key Laboratory of Chronic Disease Prevention and Therapy & Intelligent Health Management, Xinxiang, China; ^3^ Xinxiang Key Laboratory for Esophageal Cancer Imaging Diagnosis and Artificial Intelligence, Xinxiang, China

**Keywords:** contrast-enhanced computed tomography, gastric cancer, lymphovascular invasion, radiomics models, oncology

## Abstract

**Background:**

Lymphovascular invasion (LVI) is a significant risk factor for lymph node metastasis in gastric cancer (GC) and is closely related to the prognosis and recurrence of GC. This study aimed to establish clinical models, radiomics models and combination models for the diagnosis of GC vascular invasion.

**Methods:**

This study enrolled 146 patients with GC proved by pathology and who underwent radical resection of GC. The patients were assigned to the training and validation cohorts. A total of 1,702 radiomic features were extracted from contrast-enhanced computed tomography images of GC. Logistic regression analyses were performed to establish a clinical model, a radiomics model and a combined model. The performance of the predictive models was measured by the receiver operating characteristic (ROC) curve.

**Results:**

In the training cohort, the age of LVI negative (−) patients and LVI positive (+) patients were 62.41 ± 8.41 and 63.76 ± 10.08 years, respectively, and there were more male (*n* = 63) than female (*n* = 19) patients in the LVI (+) group. Diameter and differentiation were the independent risk factors for determining LVI (−) and (+). A combined model was found to be relatively highly discriminative based on the area under the ROC curve for both the training (0.853, 95% CI: 0.784–0.920, sensitivity: 0.650 and specificity: 0.907) and the validation cohorts (0.742, 95% CI: 0.559–0.925, sensitivity: 0.736 and specificity: 0.700).

**Conclusions:**

The combined model had the highest diagnostic effectiveness, and the nomogram established by this model had good performance. It can provide a reliable prediction method for individual treatment of LVI in GC before surgery.

## 1 Introduction

Gastric cancer (GC) is one of the most common malignant tumours, with a high incidence in Asia, South America and Africa. It is estimated that there are 1 million new cases of GC and 70,000 deaths globally each year (1, 2). According to the World Health Organization classification, gastric adenocarcinoma is the most common type of GC. Most gastric adenocarcinomas are thought to develop through a gradual progression from *Helicobacter* pylori-induced chronic gastritis to atrophic gastritis, intestinal metaplasia, dysplasia and ultimately adenocarcinoma (3). At present, the Lauren and Borrmann classifications are commonly used to categorise them. Furthermore, some patients with GC have both intestinal and diffuse types in the GC specimen, which is separated into mixed types (4). The widely used Borrmann classification system divides advanced GC into four types depending on its gross appearance: mass type (type 1), ulcerative type (type 2), infiltrative and ulcerative type (type 3) and diffusely infiltrative type (type 4) (5). These types can be easily defined by endoscopic morphologic assessment before preoperative therapy, as well as by gross histopathologic assessment after surgery.

Lymphovascular invasion (LVI) is defined as the lymphatic vessels and/or blood vessel invasion of malignant tumour cells within the primary tumour and surrounding tissues. Lymphovascular invasion plays an important role in cancer cell spreading and lymph node metastasis, and it is associated with an increased risk of micrometastasis (6). Studies have revealed that LVI is an independent prognostic factor for predicting clinical outcomes for patients with GC (6). Although LVI is considered to be a key prognostic factor of unsatisfactory survival outcomes in various cancers, accurate identification of LVI status before operation is still difficult, as LVI is mainly found through postoperative pathology.

Lambin et al. first put forward the concept of radiomics in 2012 (7). With high-throughput computing, it is now possible to rapidly extract innumerable quantitative features from tomographic images (computed tomography [CT], magnetic resonance [MR] or positron emission tomography [PET] images). Recent research has found that radiomics methods exhibit high accuracy and sensitivity in diagnosing GC. By analysing a variety of imaging features, researchers can more accurately locate and assess tumours. Radiomics contributes to the early detection of lesions, including tiny tumours and early-stage malignancies. This is crucial for improving treatment success rates and survival rates (8, 9) and can provide personalised diagnosis and treatment plans for each patient (9, 10). Research has found that its advantages are typically non-invasive, causing minimal harm to patients, and it is suitable for repeated monitoring and long-term follow-up. Combining different types of imaging techniques, such as CT, MRI and PET, can provide comprehensive information, aiding in a more thorough understanding of lesions and utilising computer algorithms for image analysis; this enables efficient and automated data processing, thus relieving the workload of healthcare professionals (11). However, it also has some disadvantages, such as (1) some advanced imaging techniques may lead to higher costs, limiting their widespread adoption in certain regions and healthcare systems, (2) radiomics methods face challenges in standardisation; differences in devices and methodologies across studies may affect result consistency and (3) the vast amount of medical imaging data raises privacy concerns, necessitating strict data security measures (12, 13). With its development, it has now been applied to any medical research that can image a disease or condition (14). At present, there are few studies on the use of radiomics to investigate LVI of GC. Therefore, this study aims to develop a clinical model, a radiomics model and a combination model based on contrast-enhanced CT to predict LVI of GC and to evaluate the potential clinical applicability of the model.

## 2 Materials and methods

### 2.1 Patients

The sample size calculation formula was as follows: *n* = *Z* ^2^ × *p* × (1 − *p*)/*E* ^2^, where n is the required sample size, *Z* is the Z-score corresponding to the desired confidence level (1.96 for a 95% CI), *p* is the estimated proportion of the population with a particular characteristic and *E* is the margin of error.

**Figure 1:**
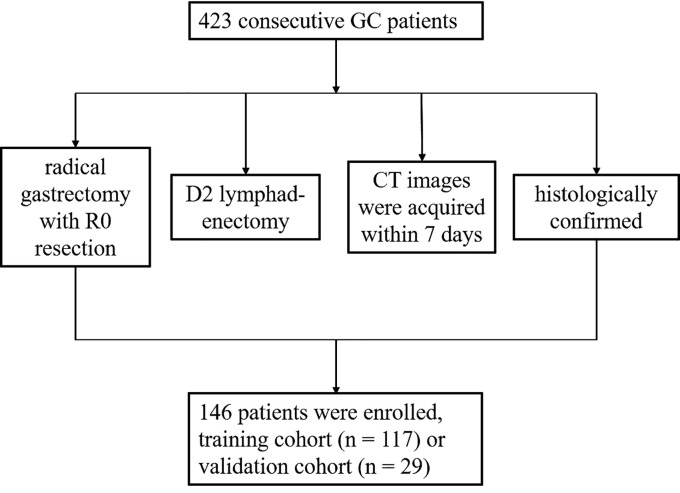
Flow chart.

This study was approved by the Institutional Review Board of our hospital. The informed consent requirement was waived. A total of 423 consecutive patients with GC at the hospital between January 2019 and May 2022 were enrolled in this retrospective study. The inclusion criteria were as follows: (1) patients received radical gastrectomy with R0 resection and D2 lymphadenectomy; (2) contrast-enhanced abdominal CT images were acquired within 7 days before the operation; (3) GC diagnosis was histologically confirmed; (4) the image quality was satisfactory for analysis. The exclusion criteria were as follows: (1) received antitumour treatment before surgery; (2) the lesion was too small to be recognised by CT; (3) combined with other malignant tumours. In total, 146 patients were enrolled. All enrolled patients were randomly allocated to either the training cohort (*n* = 117) or the validation cohort (*n* = 29) at a ratio of 8:2. The clinical factors, including age, gender, tumour diameter, differentiation status, Borrmann classification, Lauren type and the test results of four serum biomarkers, namely carbohydrate antigen 125 (CA125), carbohydrate antigen 19-9 (CA19-9), carcinoembryonic antigen (CEA) and alpha-fetoprotein (AFP), were obtained. An overview of the study’s workflow is shown in **Figure 1**.

### 2.2 Computed tomography examination

All patients were asked to fast for 8 hours, drink 800–1,000 ml of water and practice holding their breath before the CT examination. Patients were administered scopolamine hydrochloride intramuscularly before the examination to reduce gastrointestinal motility artefacts. The CT examination was performed using Revolution CT (GE Medical Systems) and Aquilion ONE CT (CANON Medical Systems) devices. All patients were in the supine position, and the scan covered the upper or the entire abdomen. The scanning parameters were as follows: tube voltage 120 kVp, 375 reference mAs with an automated tube current modulation system, slice thickness 5.0 mm, slice interval 5.0 mm, matrix 512 × 512, rotation time 0.5 s and reconstruction section thickness 1.25 mm. After an intravenous injection of contrast medium (3.0–3.5 ml/s ioversol, 320 mg/ml) via a syringe pump, the arterial phase (AP) and venous phase (VP) scans were acquired following delays of 30–35 s and 70–75 s, respectively.

### 2.3 Region of interest segmentation and feature extraction

The AP and VP images of all patients were imported into the image processing software 3D Slicer 4.11.0 (https://www.slicer.org/) in the DICOM format. Two doctors unaware of the pathology manually delineated the upper and lower consecutive three-layer regions of interest (ROIs) centred on the largest cross-section of the tumour. In case of a disagreement, the two doctors discussed with each other until they reached a consensus. Using a spline interpolation algorithm, all CT images were resampled to the same size (1 × 1 × 1 mm), regardless of the scanner from which they were acquired. A total of 1,702 radiomic features were extracted from the AP and VP CT images for each patient. These features included shape, first order, grey level co-occurrence matrix, grey level size zone matrix, grey level run length matrix, grey level dependence matrix and neighbouring grey tone difference matrix features.

### 2.4 Feature selection

To identify robust and reliable radiomics features, feature selection was performed in the following five steps. First, all the features were centralised and standardised. Second, the intra-class correlation coefficient (ICC) was calculated for the re-segmentation data. All ICC values ≥0.75 were reserved for stable features. Third, the independent sample *t*-test or Mann–Whitney U test was applied to compare the features between the LVI positive (+) group and the LVI negative (−) group for selecting the potentially important features. Fourth, the least absolute shrinkage and selection operator (LASSO) method was used to reduce the dimension. The variables were selected through the regularisation process, and the complexity was adjusted simultaneously to obtain the optimal feature subset to improve the accuracy and repeatability of the radiomics prediction model. This study tuned the regularisation parameter (λ), and tenfold cross-validation was used to select features. The best feature subset was obtained using the minimum variance model principle. Finally, the Pearson correlation coefficient of the retained features was analysed to verify whether there was a high correlation between the retained features (i.e. whether there was collinearity). The radiomics signature (RS) was built based on the selected features, and each patient’s corresponding radiomics score was calculated.

### 2.5 Model construction

The RS was built based on the selected features, and each patient’s corresponding radiomics score was calculated. Based on the selected features, the radiomics model was established by a multivariate logistic regression algorithm. The statistically significant risk variables (radiomics score and clinical factors) obtained from the univariate logistic regression analysis were then entered into the multivariate analysis to establish the clinical and combined models. A nomogram was generated for the combined model visualisation, graphical evaluation of variable importance and calculation of predictive accuracy. The Hosmer–Lemeshow test was performed to assess the goodness-of-fit of the nomogram. A calibration curve, obtained by plotting the actual LVI probability against the nomogram-predicted probability of LVI, was used to assess the calibration of the nomogram. The decision curve was used to evaluate the clinical utility of the nomogram.

### 2.6 Statistical analysis

The statistical analysis was conducted using the SPSS 25.0 and R 4.2.1 (https://www.R-project.org). A *p-*value <0.05 was set, indicating a statistically significant difference. The differences between continuous clinical variables (age, diameter, AFP, CEA, CA125 and CA19-9) were evaluated by the independent sample *t*-tests or Mann–Whitney U test; these variables were expressed as mean ± standard deviation or median and interquartile range, as appropriate. The chi-squared test was used to compare dichotomous clinical variables (gender, differentiation, Borrmann classification and Lauren type) between LVI (+) and LVI (−); these variables were expressed as numbers and percentages. Following that, univariate and multivariate logistic regression analyses were performed to identify the relationship between clinical factors and LVI status. The LASSO analysis was conducted using the ‘glmnet’ package in the R software, and the nomogram, calibration curve and decision curve were made using the ‘rms’ and ‘rmda’ packages. Diagnosis efficacy was assessed using the receiver operating characteristic (ROC) curve with the area under the curve (AUC).

## 3 Results

### 3.1 Clinical characteristics

**Table 1** lists the clinical factors of the training and validation cohort patients. A total of 146 patients were included in this study, including 82 patients with LVI (+) and 64 patients with LVI (−) Among them, there were more male (*n* = 63) than female (*n* = 19) patients in the LVI (+) group.

**Table 1:** Analysis of clinical characteristics.

Factors	Training cohort (n = 117)			Validation cohort (n = 29)		
	LVI(-)	LVI(+)	P	LVI(-)	LVI(+)	P
Gender, n (%)			0.672			0.494
Male	42(77.8)	51(81.0)		5(50.0)	12(63.2)	
Female	12(22.2)	12(19.0)		5(50.0)	7(36.8)	
Age	62.41(±8.41)	63.76(±10.08)	0.301	59.50(±10.66)	64.26(±6.79)	0.073
AFP	3.20(3.38)	3.33(3.34)	0.928	2.94 (3.15)	3.10(3.59)	0.735
CEA	3.20(2.87)	2.33(3.92)	0.166	2.23(2.03)	3.13(6.10)	0.138
CA125	11.50(9.23)	9.43(7.38)	0.226	10.32 (4.92)	12.03(8.50)	0.266
CA19-9	10.90(11.57)	11.26(15.05)	0.557	10.41 (18.69)	15.30(30.05)	0.211
Diameter	16.76(±4.84)	18.68(±5.77)	0.378	13.98(±3.78)	19.21(±7.79)	0.034
Differentiation, n (%)			0.001			0.798
well	7(13.0)	2(3.2)		1(10.0)	3(15.8)	
moderate	31(57.4)	21(33.3)		3(30.0)	7(36.8)	
poor	16(29.6)	40(63.5)		6(60.0)	9(47.4)	
Borrmann, n (%)			0. 044			0.269
I	5(9.3)	2(3.2)		1(10.0)	1(5.3)	
II	25(46.3)	19(30.2)		4(40.0)	10(52.6)	
III	18(33.3)	37(58.7)		2(20.0)	7(36.8)	
IV	6(11.1)	5(7.9)		3(30.0)	1(5.3)	
Lauren, n (%)			0.001			0.790
intestinal	34(63.0)	18(28.6)		4(40.0)	6(31.6)	
diffuse	8(14.8)	24(38.1)		4(40.0)	7(36.8)	
mixed	12(22.2)	21(33.3)		2 (20.0)	6(31.6)	

LVI (-) vascular invasion is negative, LVI (+) vascular invasion is positive, n cases.

In the training cohort, the poor differentiation rate of LVI (−) patients was higher than that of LVI (+) patients, and there was a significant difference in Borrmann classification between LVI (+) and LVI (−) (*p* < 0.05), and there was a significant difference in Lauren type between LVI (+) and LVI (−) (*p* < 0.05). Other features – sex, age, AFP, CEA, CA125, CA19-9 and diameter – had no statistical difference between groups. In the validation cohort, there was a significant difference in diameter between the two groups (*p* < 0.05), and the other factors were not statistically significant. Whether in the training or validation cohorts, the tumour diameter of LVI (+) patients was more extensive than that of LVI (−) patients.

### 3.2 Feature selection

**Figure 2:**
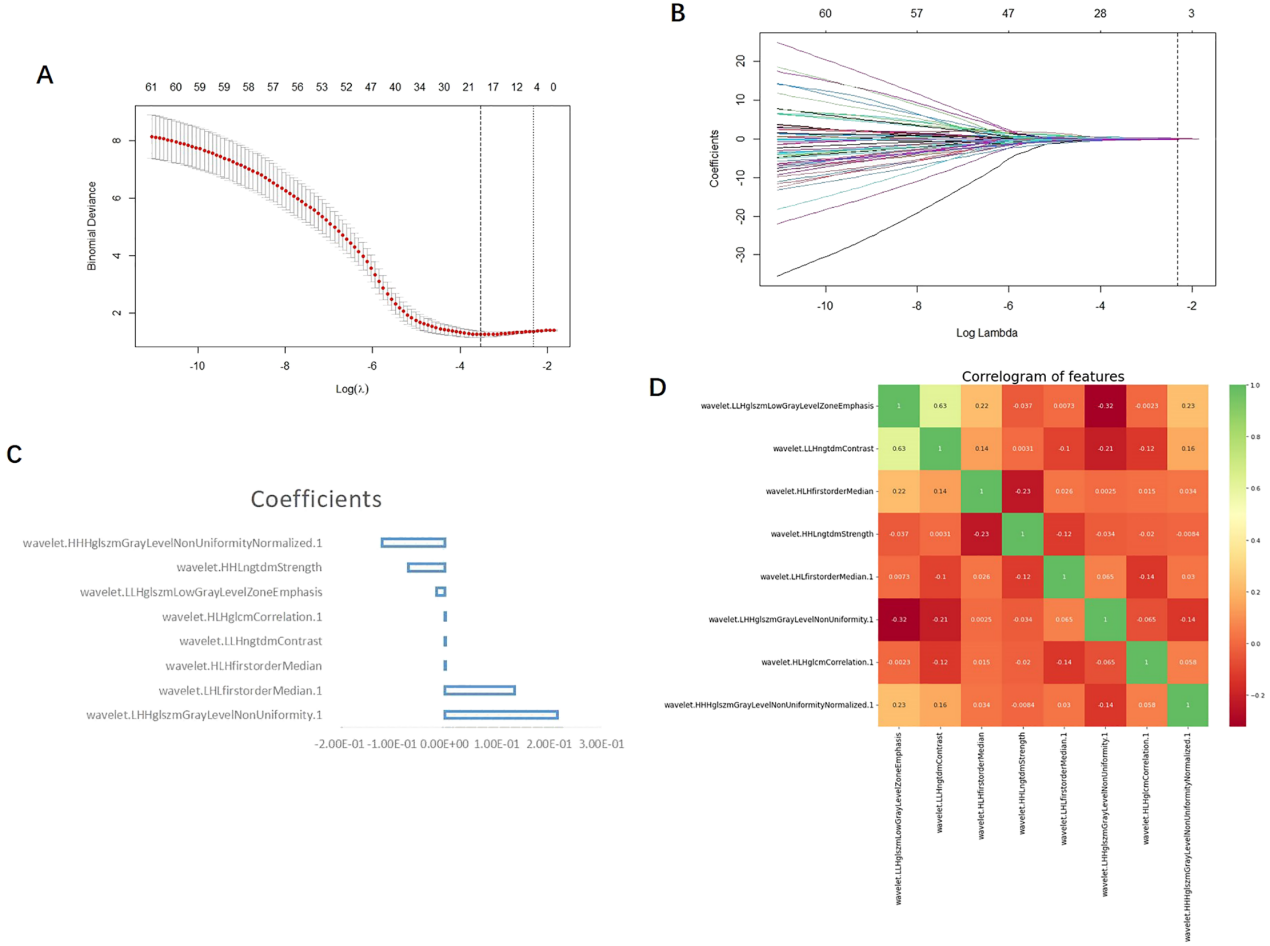
**(A)** LASSO the two vertical dotted lines in figure a represent the logarithm λ of the minimum mean square error (lambda.min) (left dashed line) and the logarithmic λ (right dashed line) of the minimum distance standard error (lambda.1se); lmabda.min is the best value, and lambda.1se is a model with excellent performance and the least number of independent variables. **(B)** Each curve in b represents the changing trajectory of each independent variable coefficient, the ordinate is the coefficient value, and the Abscissa is the number of non-zero coefficients in the model at this time. With the continuous increase of the parameters, the coefficient is finally compressed to a variable of 0, which shows that it is more important. The dotted line in the graph is the number of features under the lambda.1se parameter. **(C)** The vertical coordinate of the feature weight diagram represents the feature name, and the Abscissa represents the coefficient value; Coefficients: coefficient. **(D)** The scale on the right side of the correlation coefficient heat map shows the color depth corresponding to different correlation coefficients, and the higher the color is, the greater the correlation is; as can be seen from the figure, the maximum correlation coefficient between each feature is 0.63, so there is no highly correlated feature pair.

**Figure 3:**
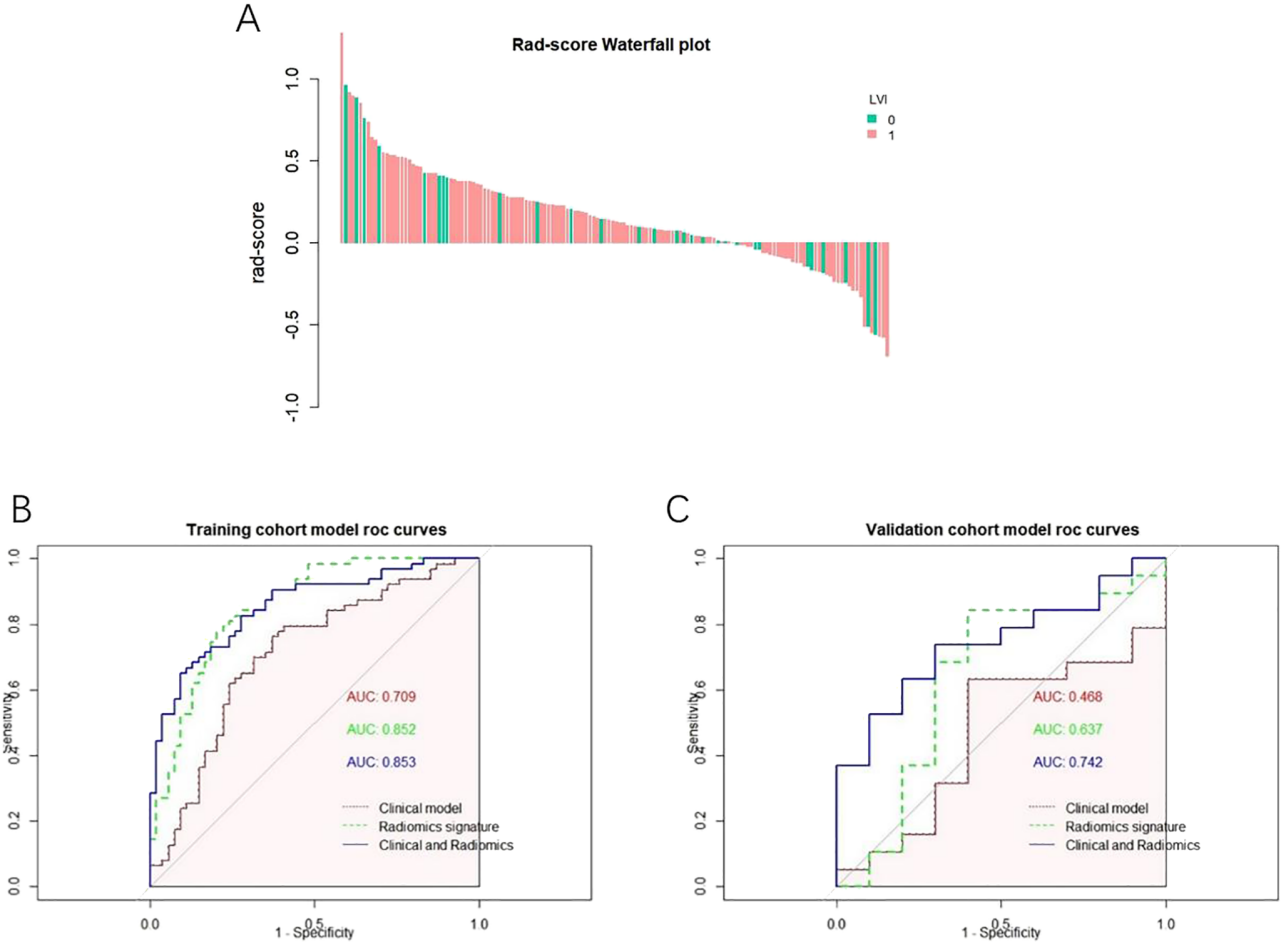
**(A)** Rad-score Falls Map Abscissa each bar represents a patient, green represents the patient of LVI (-), pink represents the patient of LVI (), and the ordinate represents Rad-score from-1 to 1. **(B)** ROC curve of the model a ROC curve of each model in the training set; **(C)** ROC curve of each model in the test set.

A total of 1,702 radiomic features were initially extracted from the segmented CT images of the GC ROI, and 1,389 features with ICC ≥0.75 were retained. After the independent sample *t*-test or Mann–Whitney U test, 97 features with *p <*0.05 were retained. To reduce dependency and redundancy, LASSO regression was used to reduce the dimensions of these features (**Figures 2A, B**). After adjusting the parameter λ by tenfold cross-validation, the optimal feature subset containing 8 features was finally obtained for model construction (**Table 2**, **Figure 2C**). After the Pearson correlation coefficient analysis, no high correlation was found between the retained features (the maximum correlation coefficient was 0.63); therefore, there was no redundant feature (**Figure 2D**). The retained 8 imaging features were calculated according to Rad-score, and finally the RS of each patient was obtained (**Figure 3A**).

**Table 2:** Feature names and weight coefficient results.

Feature	Phase	Coefficients
wavelet.LLHglszmLowGrayLevelZoneEmphasis	Arterial phase	-1.574158e-02
wavelet.LLHngtdmContrast	Venous phase	-4.279167e-09
wavelet.HLHfirstorderMedian	Venous phase	2.909216e-09
wavelet.HHLngtdmStrength	Venous phase	-6.950219e-02
wavelet.LHLfirstorderMedian.1	Arterial phase	1.330830e-01
wavelet.LHHglszmGrayLevelNonUniformity.1	Arterial phase	2.157122e-01
wavelet.HLHglcmCorrelation.1	Venous phase	-5.867018e-09
wavelet.HHHglszmGrayLevelNonUniformityNormalized.1	Venous phase	-1.189336e-01

### 3.3 Establishment of model and evaluation of diagnostic efficiency

**Figure 4:**
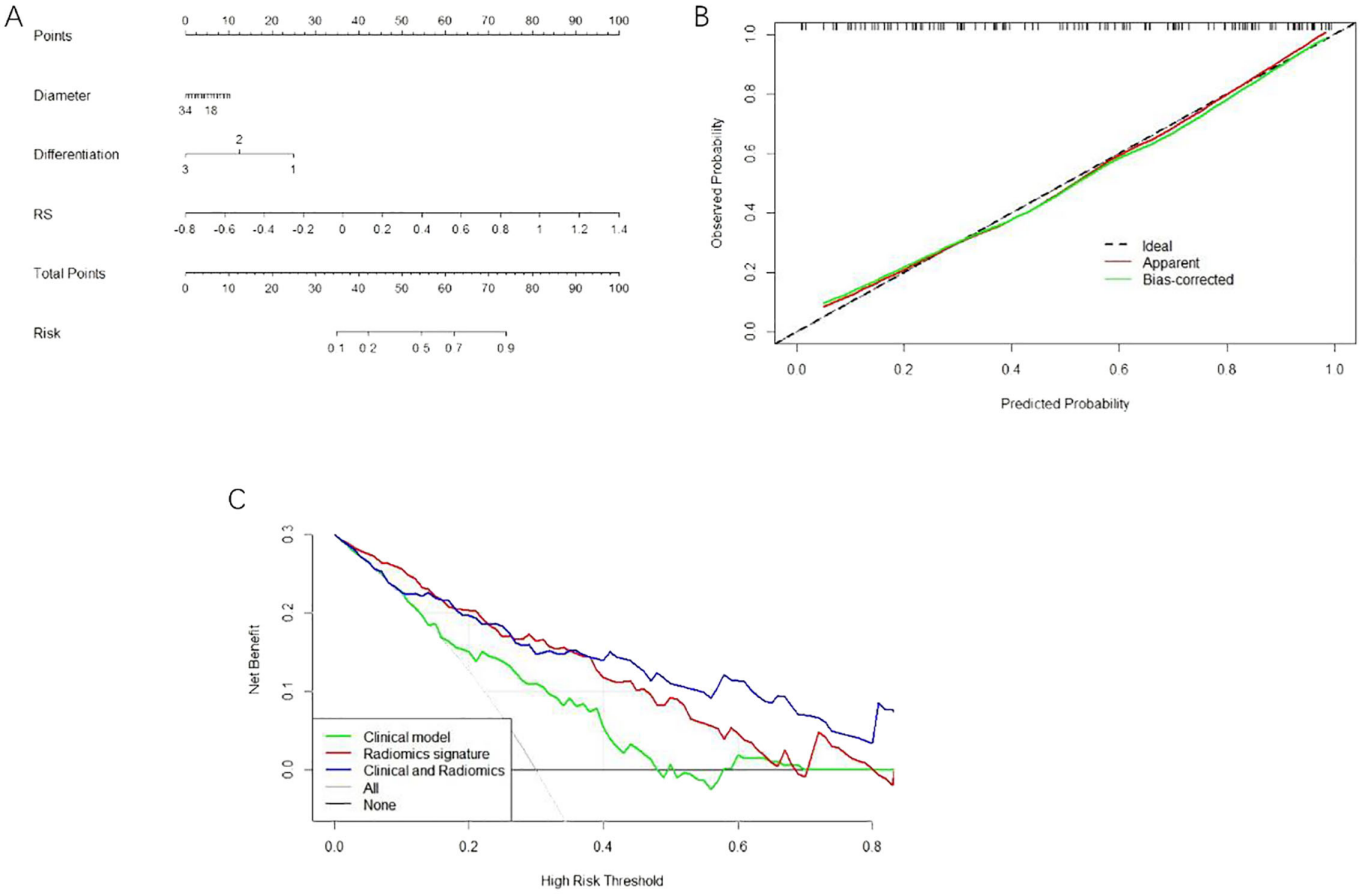
**(A)** The nomogram consists of the maximum diameter of Diameter tumour; the degree of Differentiation; and RS imaging tagging. **(B)** The calibration curve of the nomogram this chart shows that there is a high consistency between the observation and prediction results of the nomogram. **(C)** DCA decision curve validation of three model’s clinical decision-making.

Univariate and multivariate logistic regression determined that the diameter and differentiation were independent risk factors for LVI (**Table 3**), and these two variables were included in logistic regression to establish the clinical model. In addition, the radiomics model was established based on the RS by using a multivariate logistic regression algorithm, and the combined model was developed by integrating the RS and clinical independent risk factors. In the training cohort, the AUC values of the clinical model, the radiomics model and the combined model were 0.709 (95% CI: 0.613–0.805), 0.852 (95% CI: 0.783–0.921) and 0.853 (95% CI: 0.784–0.920), respectively. In the validation cohort, the AUC values of three corresponding models were 0.468 (95% CI: 0.239–0.697), 0.638 (95% CI: 0.391–0.881) and 0.742 (95% CI: 0.559–0.925), respectively. The combined model showed a higher predictive capability in the training cohort; therefore, the combined model was chosen as the final model and presented as a nomogram. The specific performances of the models are shown in **Table 4**. The ROC curves of the three models are illustrated in **Figures 3B, C**, and the nomogram is presented in **Figure 4A**.

**Table 3:** Univariate and multivariate logistic regression analysis of clinical characteristics.

Variable	Univariate Logistic Regression			Multivariate Logistic Regression		
	β	OR(95%CI)	P	β	OR(95%CI)	P
Gender	0.392	1.480(0.592-3.698)	0.401			
Age	-0.005	0.995(0.954-1.039)	0.828			
AFP	-0.003	0.997(0.992-1.002)	0.182			
CEA	0.001	1.001(0.999-1.002)	0.354			
CA125	0.001	1.001(0.991-1.011)	0.826			
CA19-9	-0.012	0.988(0.975-1.002)	0.091			
Diameter	-0.077	0.926(0.862-0.995)	0.035	0.077	1.080(1.005-1.159)	0.035
Differentiation	-0.795	0.452(0.220-0.926)	0.030	0.795	2.214(1.080-4.541)	0.030
Borrmann	0.263	1.300(0.725-2.333)	0.379			
Lauren	-0.383	0.682(0.425-1.094)	0.112			

**Table 4:** ROC diagnostic performance of the model.

	Training cohort model			Validation cohort model		
	Clinical	Radiomics	C+R	Clinical	Radiomics	C+R
AUC	0.709	0.852	0.853	0.468	0.638	0.742
95%CI	0.613-0.805	0.783-0.921	0.784-0.920	0.239-0.697	0.391-0.881	0.559-0.925
threshold	0.485	0.588	0.655	0.606	0.255	0.561
Accuracy	0.701	0.786	0.769	0.620	0.7	0.724
Sensitivity	0.761	0.777	0.650	0.631	0.842	0.736
Specificity	0.629	0.796	0.907	0.600	0.600	0.700

### 3.4 Calibration and clinical usefulness analysis

The calibration curve analysis demonstrated good agreement between prediction and observation for the nomogram (**Figure 4B**). The decision curve analysis (DCA) for the radiomics model, the clinical model and the combined model are presented in **Figure 4C**. The DCA demonstrated the effectiveness of the three models in clinical decision-making; the clinical application value of the combined model is seen to be higher than that of the radiomics model and clinical model, and it can provide better net benefits (**Figure 4C**).

## 4 Discussion

Among the clinical features, endoscopic biopsy and tumour markers have become the routine preoperative examination of GC (15, 16). However, at present, LVI is mainly found by postoperative pathology, and accurate preoperative identification of LVI status remains difficult. Radiomics can be used for GC screening, particularly for early detection in high-risk populations. Usually, the larger the tumour diameter (17) and the lower the degree of differentiation are, the higher the tumour invasiveness and the higher the risk of LVI. In a study of 2,090 patients, Fujikawan et al. (18) found that the maximum diameter of the tumour is one of the factors affecting LVI, which agrees with the findings of this study. In the study of the relationship between early GC vascular invasion and prognostic factors, Choi et al. (19) concluded that the degree of differentiation of early GC was statistically significant between the LVI (−) group and the LVI (+) group. In this study, we demonstrated that the tumour’s maximum diameter and differentiation were independent risk factors of GC LVI. This study also found that Borrmann typing is not a factor affecting the positive rate of LVI, which is contrary to the results of some studies (20, 21). The five tumour markers included in this study have no statistical significance after statistical analysis, which is different from the results of Ren (22) and Chen (23). The reason may be different research participants (i.e. different populations in different areas) or it may be caused by the small sample size of this study.

Radiomics is an emerging analytical method to improve the accuracy and sensitivity of GC diagnosis by assessing multiple features of medical images. It helps in identifying tumours early, including small or early malignancies, and is important in improving treatment efficiency and patient survival (8, 9). Although there are few studies on LVI of GC by radiomics, its application is promising. Some articles in imaging science also show that wavelet changes are helpful in imaging analysis (24–27). The combined model is established based on clinical characteristics and RS, and RS is calculated according to the RAD score. No matter the verification set or the test set, the combined model has the largest AUC value and the highest diagnostic efficiency. Therefore, by combining the maximum diameter and differentiation of the tumour with the RS, the nomogram can be established, and the calibration curve and DCA analysis can be carried out; the results of this procedure are encouraging. Li (28) collected 1,062 patients with GC based on enhanced CT imaging and established an imaging group model and a deep learning model to study the preoperative prediction of LVI status in patients with GC. Unlike the model established by logistic regression in this study, it used other machine learning (ML) models, namely random forest and support vector machine. Yardımcı et al. (29) established eight ML models based on CT to predict LVI for tubular gastric adenocarcinoma, although they did not use the logistic regression method. Meng et al. (30) included the task of predicting LVI in the multicentre comparative study of two-dimensional (2D) and three-dimensional (3D) CT imaging features of GC. The results showed that the AUC of the model established with 3D features in the training verification set was 0.618 and 0.615, which was lower than the AUC values of 0.704 and 0.677 for 2D features. Unlike the above study, this study selected the largest level of the tumour and its upper and lower layers when outlining the ROI.

Chen et al. (31) carried out a retrospective analysis of 160 patients with GC. Three predictive models were established according to the imaging characteristics of arterial, venous and biphasic images, and then three RAD scores were obtained by multivariate logistic regression analysis. Four predictive models were established by combining the three RAD scores with clinical risk factors, and a total of seven predictive models were constructed. The nomogram for predicting LVI was established. In this study, the RAD score was established by using the characteristics of biphasic images and then combined with clinical factors, the nomogram was established and the decision curve (which is not found in the above research) was analysed; the results were positive. Fan et al. (32) used different ML classifiers with enhanced CT, PET/CT and clinical variables to establish a model to predict the LVI state of GC before operation. Three-dimensional manual segmentation was used to extract imaging features from PET and VP CT images. This study aimed to extract imaging features from AP and VP images.

This study has several limitations. First, it is a single-centre retrospective study without multicentre validation; future studies require multicentre data and prospective designs to evaluate current studies. Second, the sample size of this study is small, and the resulting sample error results in the prediction efficiency of the training set being higher than that of the verification set. Third, there is no 3D drawing of the whole tumour when sketching the ROI. Some studies (33, 34) conclude that the model of drawing a 3D ROI has better performance; therefore, in future research, we should pay attention to the 3D ROI of the tumour.

## 5 Conclusion

Based on the preoperative clinical features of GC and enhanced CT images, the imaging and clinical models for predicting vascular invasion of GC and the combined model combined with imaging and clinical features were established and verified. It was found that the combined model offered the highest diagnostic efficiency. It was found that the DCA analysis result of the nomogram was better, which was beneficial in improving the clinical decision-making efficiency of patients with GC. However, experiments based on multicentre retrospective verification and prospective design of large samples of 3D interest need to be further studied and verified.

## Data Availability

The original contributions presented in the study are included in the article/supplementary material. Further inquiries can be directed to the corresponding author.
